# Predictive Ability of Perfusion Index for Determining the Success of Adductor Canal Nerve Block for Postoperative Analgesia in Patients Undergoing Unilateral Total Knee Arthroplasty

**DOI:** 10.3390/life13091865

**Published:** 2023-09-04

**Authors:** Hye Joo Yun, Joong Baek Kim, Hyun Sik Chung

**Affiliations:** Department of Anesthesiology and Pain Medicine, Eunpyeong St. Mary’s Hospital, College of Medicine, The Catholic University of Korea, Seoul 03312, Republic of Korea

**Keywords:** perfusion index, peripheral nerve block, postoperative analgesia, knee arthroplasty, ultrasonography

## Abstract

Background: The perfusion index (PI) is an objective method used to determine a successful nerve block. This study aimed to investigate the prognostic ability of the PI for a successful adductor canal nerve block (ACB) and suggest the optimal PI cut-off value for predicting a block. Methods: This study was a prospective observational study and enrolled a total of 39 patients. The patients were dichotomized into successful and inappropriate ACB groups according to the results of the sensation tests. The PI value, Pleth variability index (PVi) value, and heart rate were recorded one minute before the block, at the time of the block, and one to 30 min after the block at one-minute intervals. Delta (dPI), which was defined as the difference in PI value from the baseline (the value one minute before the block), was the primary outcome. The area under the receiver operating characteristic curve (AUROC) was calculated to determine the dPI prognostic accuracy and optimal cut-off value. Results: Successful ACB was achieved in 33 patients, while ACB was inappropriate in six patients. The dPI showed significant differences between the two groups under the time interval measured (*p* = 0.001). The dPI at 5 and 20 min showed good prognostic ability for a successful block, with optimal cut-off values of 0.33 (AUROC: 0.725, 95% CI 0.499–0.951) and 0.64 (AUROC: 0.813, 95% CI 0.599–1.000), respectively. Conclusions: The dPI is an effective predictor of successful ACB. The suggested dPI cut-off values at 5 and 20 min were below 0.33 and 0.64, respectively.

## 1. Introduction

Peripheral nerve block (PNB) is widely used as an effective multimodal analgesic method to control postoperative pain [1]. The selection of specific nerves or bundles of nerves to anesthetize the operative region and control postoperative pain by PNB depends on the surgical site. PNB, including femoral, lumbar plexus, sciatic, obturator, and adductor canal nerve block (ACB), is generally used in total knee arthroplasty (TKA) for perioperative analgesia. Femoral nerve block (FNB) is the traditional standard treatment due to its advantages, including control of postoperative pain, decreased opioid use, and reduced hospital stay [2]. However, FNB affects a motor efferent nerve associated with quadriceps muscle weakness in anterior thigh muscles, which affects early mobilization and rehabilitation compared to ACB [3]. The analgesic effect of ACB is comparable to that of FNB and preserves quadriceps muscle strength, which improves early postoperative ambulation and reduces hospital stay [4,5]. Therefore, ACB has emerged as an effective alternative to achieving TKA postoperative analgesia.

The classical methods used to confirm the motor and sensory outcomes of successful PNB are subjective, time-consuming, require patient cooperation, and are not possible to use under general anesthesia. Moreover, ACB is purely a sensory block and does not involve quadriceps muscle strength preservation, unlike FNB [6]. Thus, different objective methods have been introduced to ensure successful PNB by addressing the limitations of the classical methods.

The perfusion index (PI) was introduced as an objective method to assess successful PNB. The PI can be influenced by a sympathetic block from a local anesthetic for PNB. Local anesthetic for PNB block transmission effects in ascending and descending nerve pathways. The order of the nerve fibers affected by local anesthetic is sympathetic, sensory, and then motor fibers. The resolution or regression of the block occurs first in motor fibers, sensory, and then sympathetic [7]. A successful PNB changes the blood flow and vascular tone as a consequence of the sympathetic block [8]. The PI value represents the ratio of pulsatile and non-pulsatile blood flow in peripheral tissue, which is measured by pulse oximetry. It is changed by the intravascular blood volume, pulse pressure, and vascular elasticity of the vascular walls. Thus, it can be used to measure peripheral perfusion and sympathetic stimulation with a sensor attached to an extremity, such as a finger or a toe [9]. Briefly, sympathetic nerve blockade via a successful nerve block increases local blood flow and vasodilation and eventually increases the PI value [10]. Several studies have investigated the usefulness of the PI in establishing successful regional nerve blocks, suggesting that it can be used to determine PNB success [11,12,13,14]. However, most previous studies involved upper extremity nerve blocks. The usefulness of the PI value depending on the selective distal nerve blocked by PNB, including ACB, has yet to be reported [14,15].

Thus, the purpose of this study was to investigate the clinical significance and prognostic ability of the PI in predicting ACB success and suggest the optimal PI cut-off value to predict ACB success for postoperative analgesia in patients undergoing unilateral primary TKA with an ipsilateral ACB.

## 2. Materials and Methods

### 2.1. Study Design and Subjects

A prospective observational study was carried out involving patients who underwent unilateral elective TKA surgery with ipsilateral ACB from December 2020 to July 2021 at Eunpyeong St. Mary’s Hospital, Seoul, Republic of Korea. The study was approved by the Institutional Review Board (IRB) of our institution (PC20OISI0101) and was registered with the Clinical Research Information Service (CRIS; Identifier: KCT0005421). All enrolled patients or their next of kin (this is not an option in some countries) provided written informed consent to participate in the study.

Patients aged 19 to 80 years with an American Society of Anesthesiologists (ASA) physical status score of I or II were included and scheduled for unilateral primary TKA surgery in our hospital by a single senior surgeon. The inclusion and exclusion criteria of this study are listed in Table 1. Patients with comorbidities that cause bias in measuring the PI, such as anemia, arrhythmia, and cardiovascular disease, were excluded.

### 2.2. Administration of Adductor Canal Nerve Block

All ACBs were performed by a single senior anesthesiologist well trained in ultrasound-guided nerve blocks using the same ultrasound equipment (LOGIQ E10, GE Medical Systems Ultrasound & Primary Care Diagnostics, LLC., Wauwatosa, WI, USA). All patients underwent ACB in the supine position in a designated space known as the block room before surgery using the ACB technique described by Jenstrup et al. [16]. Following the appropriate sterile skin preparation and draping, a linear ultrasound transducer (8 to 14 MHz) was placed horizontally at the mid-thigh level, approximately halfway from the anterior superior iliac spine to the patella. The femoral vein was found, and then, the saphenous nerve was identified as a hyperechoic structure with a round cross-section located lateral to the femoral artery. The femoral artery was identified using color Doppler. A 22-gauge 50-mm insulated nerve block needle (Uni-Plex^®^NanoLine^®^, PAJUNK^®^ Holding GmBH, Geisingen, Germany) was inserted in-plane in a lateral-to-medial orientation under real-time ultrasound, bypassing the sartorius muscle, and advanced toward the femoral artery. Once the needle tip was located in the adductor canal, 1 mL of saline was injected to confirm the correct position of the needle tip. ACB was performed by administering a local anesthetic as a single-shot injection under ultrasound guidance using 20 mL of 0.375% ropivacaine hydrochloride (Naropin 0.75%, Mitsubishi Tanabe Pharma Co., Osaka, Japan). 

### 2.3. PI and PVi Assessment

The PI and Pleth variability index (PVi) were measured using a multiwavelength version of pulse oximetry (Radical-7^®^; Masimo Corporation, Irvine, CA, USA) powered by Rainbow^®^ sensors (RD Rainbow SET; Masimo Corporation, Irvine, CA, USA) as attached tapes [9]. The PI is a noninvasive measure of peripheral perfusion that is based on the ratio of the pulsatile signal to the non-pulsatile signal in a pulse oximeter. The PI can be affected by a variety of factors, including changes in peripheral vascular resistance, cardiac output, and blood volume [17]. The PVi is a measure of the variability in arterial pressure or peripheral perfusion that occurs during the respiratory cycle. PVi values can be used as an indicator of fluid responsiveness, as changes in the intravascular volume can affect the variability in arterial pressure or peripheral perfusion during the respiratory cycle [18]. 

Two individual pulse oximeters were applied to the second toe of each foot to measure the PI and PVi of both the blocked and unblocked legs. The blocked leg was used to measure the PI and PVi on the extremity with an ACB, and the unblocked leg was used to measure contralateral PI and PVi as a control. The PI and PVi were recorded one minute before ACB (baseline), at the time of injecting local anesthetics for the ACB, and every minute for 30 min after the ACB (−1, B, and 1 to 30 min, respectively). To minimize the bias associated with the PI and PVi values, we maintained a uniform environment and stabilized the patient’s position as much as possible during the measurement of all parameters.

### 2.4. Anesthetic Management

No patients received any premedication before surgery. ACBs, as well as PI and PVi measurements, were performed in a designated block room prior to entering the operating room. 

In the operating room, electrocardiography, noninvasive blood pressure, and pulse oximetry monitors were applied. Noninvasive blood pressure measurements were taken every five minutes, and measurements for electrocardiography and pulse oximetry monitors were taken continuously. Oxygen was delivered to all patients at 5 L/min via a simple mask. After preloading with 5 mL/kg of crystalloid, spinal anesthesia was obtained in the lateral decubitus position with the operating leg facing down with the knees flexed and pulled high against the abdomen or chest, assuming a fetal position. Anesthesia was delivered by 0.5% hyperbaric bupivacaine (Marcaine heavy injection, 20 mg; Mitsubishi Tanabe Pharma Co., Osaka, Japan) at 10 to 12 mg in proportion to the patient’s height using a 25 G × 90 mm spinal needle with a Quincke bevel (TaeChang Industrial Co., Chungnam, Republic of Korea) at the L3–L4 intervertebral space. All procedures were performed by a single expert anesthesiologist with at least four years of experience with spinal anesthesia. 

The anesthesiologist providing care for the patient continually assessed the intraoperative level of sedation using propofol infusion (maximum of 4 mg/kg/h) and targeted a moderate level of sedation defined by the Observer’s Assessment of Alertness/Sedation score [19]. Intravenous midazolam at 0 to 3 mg was available intraoperatively to achieve this goal, and no other intraoperative opioids or sedatives were to be used. Phenylephrine and ephedrine were available at the discretion of the anesthesiologist in the event of hemodynamic changes with no specific hemodynamic targets.

### 2.5. Additional Postoperative Analgesia

Patient-controlled analgesia (PCA, AutoMed 3200, ACE Medical, Seoul, Republic of Korea) was applied to all patients with the same regimen. Fentanyl citrate (Daihan Fentanil Inj., Dai Han Pharm. Co., Seoul, Republic of Korea) at 1200 mcg was applied for PCA with 0.6 mg of ramosetron hydrochloride mixed with 0.9% normal saline at a total volume of 100 mL set to a 1 mL bolus dose (10 mcg of fentanyl) with a 10-min lockout time and no basal infusion.

### 2.6. Outcome Assessments

To confirm a successful ACB, pinprick and cold sensation tests were performed on the anteromedial side of the knee 30 min after the block. The pinprick test was performed first and involved the application of a sharp or pointed object to the skin to assess sensation. The cold test was performed separately and involved the application of a cold stimulus to the skin to assess sensation. Patients who reported a blunt pinprick and cold sensation after the sensation tests represented successful ACBs. The patients who did not feel any sensory difference represented inappropriate ACBs. 

Following inclusion in the study, the patients were provided information about the numeric rating scale (NRS) used to investigate postoperative pain. Postoperative pain was evaluated in the post-anesthesia care unit (PACU) after surgery and six and 12 h after surgery using NRS, which assessed the pain severity at that point in time on a scale of 0 to 10 [20]. 

The dPI was calculated as the difference in the PI value from the value obtained one minute before local anesthetic injection (baseline). We used the dPI instead of the PI as the primary outcome because of variations in the baseline PI values. The optimal dPI cut-off value over specific time points was used to determine a successful block. The prognostic accuracy of the PI to predict a successful nerve block was determined at different time points. Heart rate (beats/min; HR) and PVi were also used to determine dHR and dPVi, respectively, due to the variability in the dPI values. The dHR and dPVi values were calculated based on differences between HR and PVi compared to the baseline, which was the value at one minute before local anesthetic injection for the ACB.

### 2.7. Statistical Analysis 

In a preliminary study, the difference in the mean ± standard deviation (SD) of the dPI was 1.5 ± 2.963 in the blocked and unblocked legs during FNB. Based on a power of 80% and an alpha error of 0.05, we calculated that 33 subjects were required to detect this degree of difference between the blocked and unblocked legs. Thus, with a 10% dropout rate, 37 subjects were needed to enroll in this study.

All statistical analyses were conducted using R version 4.2.2 (R Foundation for Statistical Computing, Vienna, Austria). The normal distribution of the data was tested with the Shapiro–Wilk test. Continuous variables were presented as the mean ± SD and categorical variables as numbers with percentages. The continuous variables were compared using the Student’s *t*-test or Mann–Whitney *U* test as appropriate, depending on the normal distribution of the data. The categorical variables were compared using the chi-square test or Fisher’s exact test as appropriate, depending on the group size. Sequential changes over the time points were compared using repeated measures analysis of variance (rMANOVA) with Bonferroni correction as the post hoc test. Receiver operating characteristic (ROC) and area under the ROC (AUROC) curves were drawn using data obtained from successful and inappropriate ACBs to determine the extent and prognostic ability of the dPI to predict the successful ACB at each time point. The optimal cut-off value, sensitivity, specificity, positive predictive value, and negative predictive value were calculated at significant time points based on the AUROC. A paired *t*-test was performed to compare successful and inappropriate ACBs at each time point. All tests were two-sided, and a *p*-value of <0.05 was considered statistically significant.

## 3. Results

**Figure 1:**
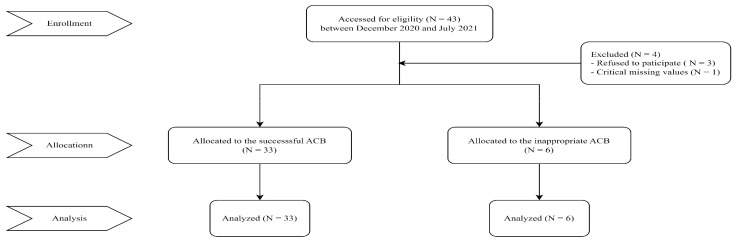
Flow diagram of the study. ACB, adductor canal nerve block.

A total of 43 patients were eligible to participate in the study. Of these, 39 patients were enrolled, excluding 3 patients who did not wish to participate and 1 patient who was missing critical data, which were related to a technical error in the PI and PVi measurements (Figure 1). The patients’ characteristics are shown in Table 2. A total of 33 (85%) patients reported successful ACB, while 6 (15%) patients who did not feel any sensory difference 30 min after the procedure represented inappropriate ACB. Seven patients with ASA I and twenty-six patients with ASA II had successful ACB. Of the patients with ASA II, 19 had hypertension, and 7 had diabetes mellitus (DM). There were no ASA I patients, and six ASA II patients were in the inappropriate ACB group. Three patients had hypertension, and one patient had DM. Among the 21 patients who had hypertension, 19 patients were taking medication combinations of an angiotensin receptor blocker (ARB) with a calcium channel blocker (CCB), 10 patients were taking ARBs, and 3 patients were taking CCBs. No statistically significant differences were detected in patient characteristics between the two groups (Table 2). 

**Figure 2:**
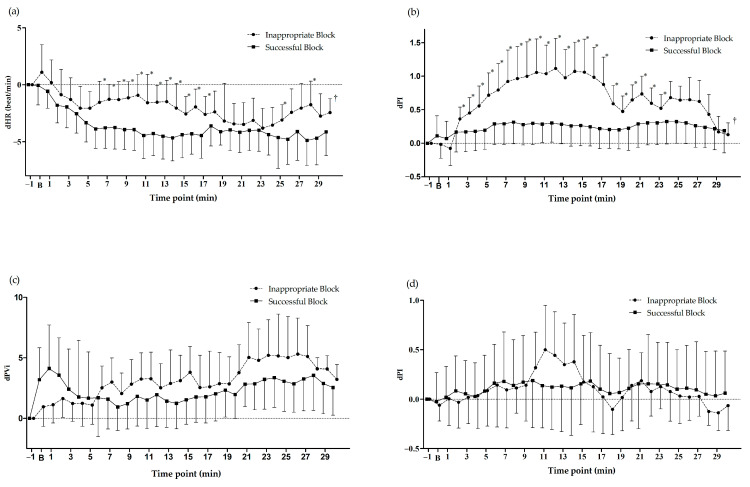
Changes in the perfusion index, Pleth variability index values, and HR at different time points. (**a**) HR, (**b**) PI values on the ipsilateral side of the ACB, (**c**) PVi values on the ipsilateral side of the ACB, and (**d**) PI values on the contralateral side of the ACB. PI, perfusion index; PVi, Pleth variability index; dPI, difference in the PI value relative to the baseline (−1 of the time point, one min before the block); dPVi, difference in the Pvi value relative to the baseline (−1 of the time point, one min before the block); dHR, difference in the HR relative to the baseline (−1 of the time point, one min before the block); B, the time of the local anesthetic injection; ACB, adductor canal nerve block. * *p* < 0.05 at the same time point between the successful and inappropriate ACBs; ^†^ *p* < 0.05 over the time points between the successful and inappropriate ACBs.

The dPI and dHR values were significantly different between the successful and inappropriate ACBs over time (*p* = 0.001 and *p* < 0.001, respectively) (Figure 2a,b). When the two groups were compared at the same time point, the dPI values were significantly different from time points 2 to 23 (*p* < 0.05), and the dHR showed significant differences from time points 6 to 17, 25, and 28 between the two groups (*p* < 0.05). The dPI value in the inappropriate ACB group was higher than in the successful ACB group. The dHR decreased less in the inappropriate ACB group than in the successful ACB group. However, the PVi and contralateral ACB dPI values were not significantly different between the two groups (*p* = 0.062 and *p* = 0.561, respectively; Figure 2c,d). 

Subsequently, the dPI at both the 5- and 20-min time points showed a good prognostic ability to predict successful blockades (Table 3). The AUROC for the dPI values at the 5- and 20-min time points was 0.725 (95% confidence interval (CI) 0.499–0.951) at a cut-off value of <0.33 and 0.813 (95% CI 0.599–1.00) at a cut-off value of <0.64. The dPI sensitivity and specificity were 81.2% and 60.0% at 5 min and 96.9% and 60.0% at 20 min (Table 4).

NRSs for postoperative pain were not statistically significant differences in the PACU immediately, 6 and 12 h after surgery between the successful and inappropriate ACB (Table 5). The amount of PCA administered over 12 h after surgery was not significantly different between the successful and inappropriate ACB (1.8 (1.1–3.25) mL vs. 2.5 (1.4–3.6) mL; *p* = 0.326). None of the participants experienced any complications or adverse effects due to ACB.

## 4. Discussion

The main findings in this study were that the PI values and HR were affected due to pain in cases of inappropriate ACB following the inadequate or inappropriate injection of local anesthetic. Thus, no significant changes in the dPI during ACB could predict successful ACB. The dPI values at 5 and 20 min (early and late, respectively) after the block were good predictors of successful ACB. However, ACB showed effective postoperative pain control in both successful and inappropriate ACBs, suggesting that the ACB provided an adequate analgesic effect in patients undergoing TKA, although the local anesthetic was not adequately injected into the adductor canal during the ACB.

Selective distal nerve blocks, including ACBs, facilitate early ambulation and discharge by minimizing motor nerve blocks, thereby reducing hospital stays and eventually minimizing hospital costs [21,22]. Several published TKA surgery studies reported that ACB was superior to FNB [23,24,25,26]. The ACB is a purely sensory block, and motor blockade by ACB affects only the vastus medialis but not the quadriceps muscle [27]. Therefore, selective distal nerve blocks are more effective than nerve plexus blocks. A selective nerve block in various dermatomes during surgery, sparing motor nerves, is desirable for both patients and clinicians [28].

Previous studies showed that the PI is a useful parameter to predict successful PNB. However, most studies focused on nerve bundles such as the brachial plexus but not distal nerve branches, such as the radial, median, and ulnar nerves, which are branches of the brachial plexus [11,12,13]. We assumed that changes in the PI values from a distal nerve block due to vasodilation via a nerve blockade would be similar to those of a plexus nerve block, even if the distal nerve block affected a smaller area. However, in this study, increased PI values were unexpectedly observed in patients with inappropriate ACBs, without significant changes in patients with successful ACBs. We speculated that the reason for no significant change in the PI values in patients with successful ACBs was as follows. First, the area of the sympathetic block by local anesthetic for ACB was too small to change the PI values, unlike regional anesthesia or a bundle nerve block, including a brachial plexus block, which blocks the whole upper or lower extremity. Second, the location of the PI measurement was not the area governed by the saphenous nerve, which was a toe, a limb extremity. Thus, it was considered that the ACB could not induce enough blood flow to the distal extremities to cause changes in the PI.

In addition, the change in HR was less in successful ACBs than in inappropriate ACBs, indicating that HR was higher in cases of inappropriate ACBs with poor nerve blocks at the same time point. Accordingly, if the local anesthetic was injected at an inappropriate location during the nerve block, such as outside a neurovascular bundle or the surrounding tissues, including muscles, the change in the HR may not decrease in an inappropriate ACB to the same extent as in a successful ACB. Moreover, in cases where local anesthetic (20 mL, a large volume compared to the structure of the injection target) was not properly injected into the adductor canal according to the ultrasound image (injected outside the structure, including muscles), the patients complained of pain or furrowed their eyebrows due to pain arising from the site of the local anesthetic injection. Thus, pain may be attributed to the inappropriate injection of local anesthetic in an inappropriate ACB, which was associated with large differences in the PI values and not decreases in the HR. This was supported by previous studies reporting PI values in response to pain [29]. Accordingly, it is possible to predict whether the PNB of a distal nerve was adequate based on the PI value. However, the detection of a successful distal nerve branch block was based on changes in the PI values due to pain by the inappropriate injection of a local anesthetic rather than changes in the PI values due to the vasodilating effect of a successful nerve block. Thus, it is possible to predict whether PNB involving the distal nerve is properly established by observing no significant changes in the PI values.

The PI values were also assessed as a predictive indicator for a regional block under general anesthesia [30,31], because it is very difficult to assess the quality of analgesia by the classic methods under general anesthesia. However, general anesthesia can affect the PI values by vasodilation [32]. Most general anesthetics, including propofol, induce a vasodilating effect by increasing the vasodilatation threshold and decreasing the vasoconstriction threshold [33]. Nevertheless, the PI values can identify the onset of a successful caudal or epidural block of the lower extremities much earlier and more consistently than either HR or mean arterial pressure. It is presumed that PI changes could be significant, even under general anesthesia, because regional anesthesia, such as caudal or epidural blocks, induces a sympathetic block in a wide area compared to a selective distal nerve block such as ACB, which induces a sympathetic block in a small area.

Because of the high variability of the PI values, we used the differences in the PI values from the baseline rather than the absolute values, and the difference was more intuitive than the ratio [12,34]. PI values may vary depending on the instrument used. However, the dPI can be expressed as a constant, because it is the difference in the values from the baseline and each time point. In a previous study related to the PI, the PI values were measured at relatively wide intervals (5 to 10 min), while we measured the PIs at shorter intervals (one minute) [35,36,37] and confirmed that the PI values at 5 and 20 min represented the early and late stages of altered PI values based on the AUROC analysis. We recommend the dPI 5 and 20 as indicators of a successful block. A dPI 5 of <0.33 and 20 of <0.46 in our study were useful cut-off values to predict a successful ACB. However, further studies are needed to validate the predictive cut-off values of dPI 5 and 20.

Additionally, the blockade of small nonmyelinated sympathetic nerve fibers by local anesthetics increased the local temperature caused by vasodilation following an increased blood flow. Thus, thermography studies could evaluate the success of a PNB by measuring the distal skin temperature [38]. Several studies evaluated changes in the thermographic values in the upper extremities after PNB [39,40,41]. Measuring the distal skin temperature has the advantage of an easy assessment but has several limitations as a predictor of successful PNB. The distal skin temperature undergoes great fluctuations due to changes in the environmental temperature, sympathetic nerve activity (e.g., stress or anxiety), local inflammation, and previous trauma. None of the previously published studies reported a thermographic study related to the lower extremities, including a selective distal nerve block. Thus, we propose a thermographic study of selective distal nerve blocks on lower extremities in future research.

The study had several limitations. First, we did not validate the PI values as an objective indicator of a successful PNB. However, the dPI at 5 and 20 min served as a surrogate endpoint and a useful clinical indicator. The 95% CI values were wide for all the observed time points, despite the statistical significance observed at 5 and 20 min in the AUROC analysis. A wider confidence interval indicates a greater uncertainty in the parameter estimations. Thus, clinical validation of our suggested cut-offs at 5 and 20 min is needed in a well-designed study with a large sample size. Second, the study included patients with hypertension and DM. Antihypertensive drugs, such as β-blockers and CCBs, or DM neuropathy, a complication of DM, have the potential to affect the sympathetic nervous system. We excluded patients taking β-blockers, and CCBs were used frequently by hypertensive patients. However, the effect of CCBs on the sympathetic nervous system was considered to be minimal [42]. Thus, patients taking CCBs were not excluded from the study. DM neuropathy is a complication that occurs when diabetes is exacerbated and progresses. For this reason, the study was conducted by limiting patients to those with ASA physical status I or II with no or well-controlled underlying diseases to minimize the influence of hypertensive medication or DM complications as much as possible. Third, ACBs were performed by a single shot of local anesthetic rather than continuous catheter-based ACB. Both single-shot and continuous catheter-based ACBs are usually used as components of a multimodal analgesia regimen following TKA [43]. Single-shot ACB for TKA has an analgesic effect over 48 h after surgery and is not inferior to continuous catheter-based ACB, suggesting technical ease and safety [44]. Fourth, the Rainbow^®^ sensor was applied to the second toe to measure the PI and PVi values. The second toe does not correspond to the territory of the saphenous nerve, which is affected by the ACB. However, it is impossible to apply the Rainbow^®^ sensor to the territory of the saphenous nerve, because it measures the PI and PVi values based on the difference in absorbance by penetrating light through tissue as attached tape. Changes in the PI and PVi values by the nerve block were presumed to result from blockage of the sympathetic nerve, unlike the affected dermatome of the nerve block. These changes in blood flow to the territory of the saphenous nerve could affect blood flow in the toes, which are the extremities of the limb used to measure the PI and PVi values. Thus, we applied the Rainbow^®^ sensor at the second toe to measure the PI and PVi values in this study. Lastly, the postoperative NRSs measured immediately after surgery and 6 and 12 h after surgery revealed no statistically significant differences between the successful and inappropriate ACBs. Our results showed adequate postoperative analgesic effects following the ACBs in patients undergoing TKA, even in cases involving the inappropriate injection of local anesthetic around the adductor canal, in which the dPI values at 5 and 20 min were above the threshold (>0.34 and >0.64, respectively). We assume that the injected local anesthetic was eventually spread effectively to the adductor canal in patients with inappropriate ACBs. Thus, the ACB was effective in both appropriate and inappropriate injections of local anesthetic as long as the local anesthetic was injected close to and inside the adductor canal for pain control in patients undergoing TKA. The strengths of the study include TKA surgery performed by a single surgeon and an ACB accomplished by a single anesthesiologist using the same ultrasound equipment.

## 5. Conclusions

In conclusion, the results of this study showed that a sympathetic block by an ACB did not significantly change the PI values. However, significant changes in the PI values resulted from pain caused by the inappropriate injection of local anesthetic against the surrounding tissues during the ACB. Thus, no significant changes in the PI values during the ACB could predict a successful ACB, and conversely, a significant change in the PI values during the ACB would mean an inappropriate injection of local anesthetic. Thus, the PI value can be used as a useful indicator for evaluating a successful ACB as a type of selective distal nerve block. The suggested optimal cut-off values associated with changes in the dPI at 5 and 20 min after the ACB for the early and late prediction of a successful ACB were <0.33 and <0.64, respectively.

## Figures and Tables

**Table 1:** Inclusion and exclusion criteria of the study.

Inclusion Criteria	Exclusion Criteria
Planned unilateral total knee arthroplasty	Unwillingness to participate in the study
ASA physical status classification score of I or II	General anesthesia
Spinal anesthesia	Known allergy to local anesthetics
	Contraindications for the application of ACB
	Localized infection
	Neurological disease in the lower extremity
	History of cardiovascular disease
	Arrhythmia
	Anemia
	Medication: β-blocker, SSRI
	Insufficient cooperation for NRS and pinprick test

ASA, American Society of Anesthesiologists; ACD, adductor canal nerve block, NRS, numeric rating scale; SSRI, selective serotonin reuptake inhibitor.

**Table 2:** Characteristics of the study population.

	Successful ACB (N = 33)	Inappropriate ACB (N = 6)	*p*-Value
Age	68.9 ± 5.1	71.7 ± 6.4	0.253
Gender (female/male)	30 (90.9)/3 (9.1)	5 (83.3)/1 (16.7)	0.585
ASA (I/II)	7 (21.2)/26 (78.8)	0 (0.0)/6 (100.0)	0.224
Height	153.03 ± 6.44	154.71 ± 5.78	0.555
Weight	61.29 ± 9.53	65.85 ± 6.51	0.270
BMI	26.16 ± 3.54	27.57 ± 3.10	0.369
Duration of surgery (min)	63.5 ± 10.2	59.3 ± 7.7	0.348

Data are expressed as the number (proportions) of subjects or mean ± standard derivation (SD). ACB, adductor canal nerve block; ASA, American Society of Anesthesiologists physical status; BMI, body mass index.

**Table 3:** Area under the dPI receiver operating characteristic curve over time.

Time Point	AUROC	95% CI	*p*-Value	Time-Point	AUROC	95% CI	*p*-Value
−1	0.413	0.072–0.753	0.534	15	0.719	0.417–1.000	0.120
B	0.556	0.233–0.879	0.689	16	0.713	0.396–1.000	0.131
1	0.650	0.408–0.892	0.286	17	0.688	0.364–1.000	0.183
2	0.556	0.237–0.875	0.689	18	0.675	0.366–0.984	0.214
3	0.594	0.292–0.895	0.505	19	0.750	0.513–0.987	0.076
4	0.706	0.410–1.000	0.090	20	0.813	0.599–1.000	0.026 *
5	0.725	0.499–0.951	0.043 *	21	0.638	0.285–0.990	0.328
6	0.656	0.321–0.992	0.267	22	0.656	0.322–0.991	0.267
7	0.594	0.264–0.923	0.505	23	0.638	0.294–0.981	0.328
8	0.663	0.373–0.952	0.248	24	0.631	0.273–0.990	0.351
9	0.644	0.306–0.982	0.307	25	0.625	0.259–0.991	0.374
10	0.644	0.298–0.990	0.307	26	0.638	0.305–0.970	0.328
11	0.681	0.333–1.000	0.198	27	0.638	0.285–0.990	0.328
12	0.719	0.408–1.000	0.120	28	0.600	0.262–0.938	0.477
13	0.675	0.321–1.000	0.214	29	0.588	0.251–0.924	0.534
14	0.713	0.396–1.000	0.131	30	0.638	0.283–0.992	0.328

B, time of the local anesthetic injection; dPI, difference in the PI value relative to the baseline (−1 of the time point, one min before the block); AUROC, area under the receiver operating characteristic curve; CI, confidence interval. * *p* < 0.05.

**Table 4:** Comparison of the dPI predictive values, sensitivity, specificity, diagnostic accuracy, and differences in the AUROCs between successful and inappropriate ACBs 5 and 20 min after the procedure in predicting a successful adductor canal block for postoperative pain control in patients undergoing knee surgery.

Time Point	Threshold Score	Sensitivity	Specificity	PPV	NPV	Diagnostic Accuracy	ΔAUROC	95% CI	*p*-Value
5	<0.33	81.2	60.0	92.9	33.3	0.725	Ref.	0.499–0.951	Ref.
20	<0.64	96.9	60.0	93.9	75.0	0.813	0.088	0.599–1.000	0.125

dPI, difference in the PI value relative to the baseline (−1 of the time point, one min before the block); PPV, positive predictive value; NPV, negative predictive value; CI, confidence interval; AUROC, area under the receiver operating characteristic curve; ΔAUROC, difference in the AUROCs.

**Table 5:** NRSs for evaluating postoperative pain intensity.

	Successful ACB (N = 33)	Inappropriate ACB (N = 6)	*p*-Value
PACU	0.21 ± 0.93	0.31 ± 0.88	0.809
Post-operation 6 h	1.39 ± 0.86	1.33 ± 0.82	0.874
Post-operation 12 h	1.52 ± 1.06	1.33 ± 0.82	0.694

Data are presented as the mean ± standard derivation. ACB, adductor canal nerve block; NRS, numeric rating scale; PACU, post-anesthesia care unit.

## Data Availability

The datasets generated and/or analyzed during the current study are not publicly available, because disclosing patients’ personal information is against the law; however, deidentified datasets are available from the corresponding author upon reasonable request.
